# AKI in Patient with Acute Viral Hepatitis

**DOI:** 10.34067/KID.0000000000000215

**Published:** 2023-11-30

**Authors:** Vishal Ramteke, Sagar Patil, Dipak Nandurkar

**Affiliations:** 1 Department of Nephrology, Alexis Hospital, Nagpur, Maharashtra, India; 2 Department of Gastroenterology, Alexis Hospital, Nagpur, Maharashtra, India; 3 Department of Internal Medicine, Alexis Hospital, Nagpur, Maharashtra, India

**Keywords:** AKI, clinical nephrology, hemodialysis, kidney biopsy, kidney tubule

## Abstract

**Figure:**
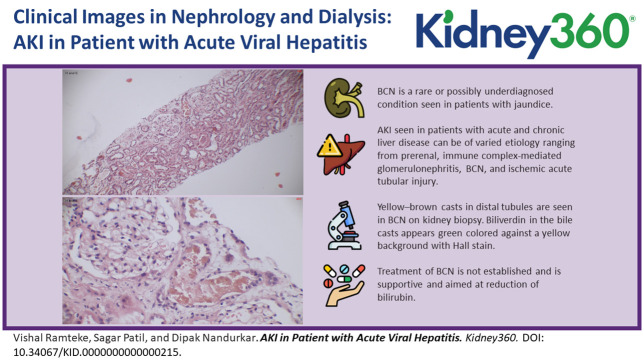

## Case Description

**Figure 1:**
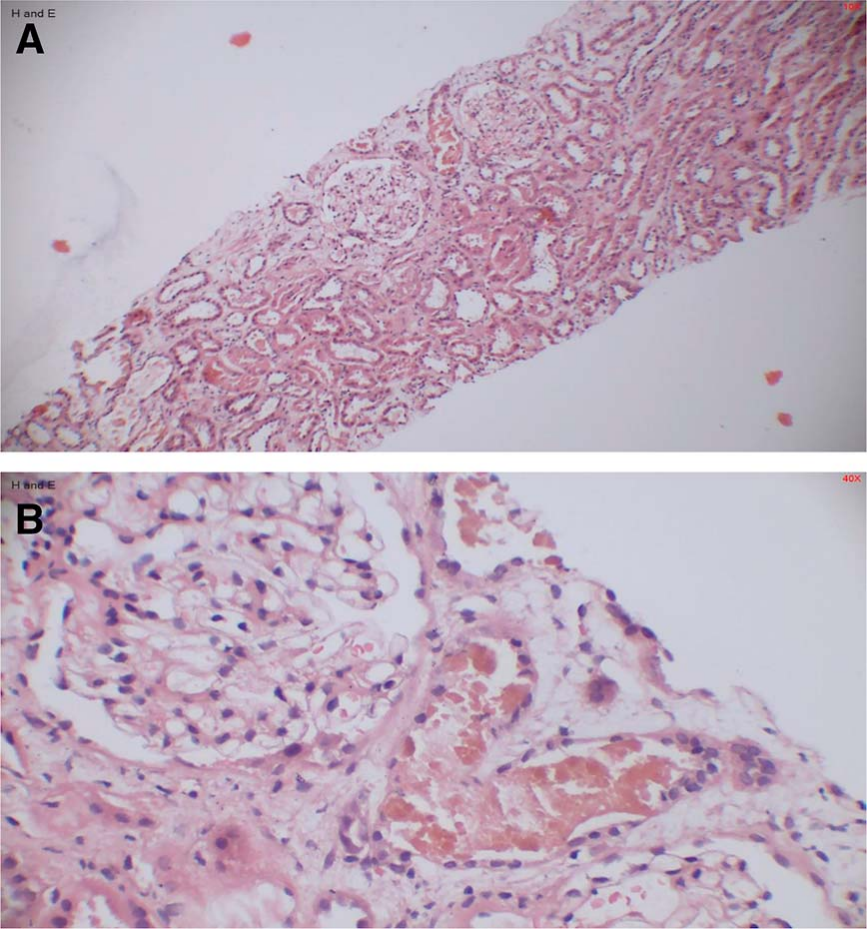
**Kidney biopsy showing intratubular bile cast.** (A) Microphotograph showing normal glomeruli without crescents. There is tubular injury with few dilated tubules showing casts (H&E, ×10 magnification). (B) Microphotograph showing an injured dilated tubule with pigmented golden–brown granular cast material suggestive of bile cast (H&E, ×40 magnification).

A 34-year-old man presented to emergency department with 4 days of vomiting, decreased appetite, and yellowness of eyes and urine. On physical examination, he had icterus with signs of dehydration and mild right upper quadrant tenderness. Investigation showed white blood cell count of 13,200/mm^3^ (3500–10500/mm^3^), total bilirubin of 11.2 mg/dl (<1.1 mg/dl), direct bilirubin of 9.9 mg/dl (<0.2), aspartate transaminases of 7400 U/L (<32 U/L), alanine transaminase of 5414 U/L (<33 U/L), blood urea of 160 mg/dl (6–20 mg/dl), serum creatinine of 5.7 mg/dl (0.7–1.2 mg/dl), and international normalized ratio of 1.6 (0.8–1.2). Urinalysis showed trace proteinuria and urobilinogen, 10–12 red blood cells per high-power field, and granular casts. His ultrasound of the abdomen showed hepatomegaly with normal sized kidneys. Acute viral hepatitis was diagnosed based on serology which was positive for hepatitis A (Immunogloulin M). The patient was started on intermittent hemodialysis due to oliguria and AKI associated with worsening with progressive jaundice. He received ursedeoxycholi acid 300 mg twice a day for cholestasis with supportive therapy. The patient continued to be oliguric for more than 3 weeks. Complement levels, antinuclear antibody, and antineutrophil cytoplasmic antibody performed to rule out any immune-mediated kidney injury were normal. He was subjected to ultrasound-guided kidney biopsy which showed severe acute tubular injury with golden brown pigmented cast in tubular lumen and normal immunofluorescence (Figure 1, A and B). The kidney biopsy findings were suggestive of acute tubular injury with bile cast nephropathy (BCN). The patient was started on prednisolone 30 mg per day which was gradually tapered off. The patient improved over next two weeks and did not require dialysis after 2 weeks. The patient has residual kidney disease, and his serum creatinine on follow-up is 1.3 mg/dl.

## Discussion

BCN is one of the multiple causes of AKI in patients with acute viral hepatitis. It was first described in 1899 by Qunicke, and similar cases have been reported worldwide.^1^ Elevated bilirubin levels leading to oxidative injury to renal tubules, direct tubular epithelial cells injury by sulfated bile salts, bile cast deposition leading to tubular, negative inotropic, and chronotropic effects in jaundiced patient leading to renal hypoperfusion are putative mechanisms for AKI.^2,3^ High levels of bilirubin (more than 20 mg/dl) or rapidly increasing bilirubin levels are considered risk factors for BCN. The pathology is limited to distal tubules, which show denuded tubular epithelium, luminal dilatation with yellow–brown bile casts. Hall stain is used to confirm the presence of bile in the tubular casts. It contains Fouchet reagent (trichloroacetic acid), which converts bilirubin into biliverdin giving green color against a yellow background.^4^ The color ranges from olive green to emerald green, depending on the concentration of bilirubin.

BCN is rare, and there are no established treatment guidelines. Interventions to relieve biliary obstruction *via* endoscopic retrograde cholangiopancreatograph with stent placement have helped to improve outcome in cases with obstructive jaundice. Extracorporeal therapies, such as plasmapheresis, molecular adsorbents recycling system, single-pass albumin dialysis, and coupled plasma filtration adsorption, have shown to improve survival rates in some patients with acute liver failure.^1–3^ Medical therapy with steroids, ursodeoxycholic acid, and lactulose have shown minimal benefit.

## Teaching Points

- BCN is a rare or possibly underdiagnosed condition seen in patients with jaundice.
- AKI seen in patients with acute and chronic liver disease can be of varied etiology ranging from prerenal, immune complex-mediated glomerulonephritis, BCN, and ischemic acute tubular injury.
- Yellow–brown casts in distal tubules are seen in BCN on kidney biopsy. Biliverdin in the bile casts appears green colored against a yellow background with Hall stain.
- Treatment of BCN is not established and is supportive and aimed at reduction of bilirubin.

## Disclosures

All authors have nothing to disclose.
